# End-of life medical spending and care pathways in the last 12 months of life: A comprehensive analysis of the national claims database in France

**DOI:** 10.1097/MD.0000000000034555

**Published:** 2023-08-04

**Authors:** Arnaud Nze Ossima, Daniel Szfetel, Bénédicte Denoyel, Omar Beloucif, Joelle Texereau, Louis Champion, Jean François Vié, Isabelle Durand-Zaleski

**Affiliations:** a Semeia, Paris, France; b AP-HP Health Economics Research Unit, Hotel Dieu Hospital, Paris, France; c Visiatio - Voisins and Soins, Paris, France; d Fédération des Prestataires de Santé à Domicile (FEDEPSAD), Paris, France; e AP-HP Service de Physiologie-Explorations Fonctionnelles, Hôpital Cochin, Université de Paris, Paris, France; f INSERM UMR 1153 CRESS, Clinical Epidemiology (Methods) Research Team, Paris Descartes University, Paris, France; g Université Paris Est Créteil, Créteil France.

**Keywords:** Alzheimer, cancer, care pathways, claims, end of life, expenditures, medical spending

## Abstract

To inform policy makers on efficient provision of end-of-life care, we estimated the 12-month medical expenditures of French decedents in 2015.

**Methods:**

We estimated total medical expenditures by service type and diagnosis category, and analyzed care pathways for breast cancer, dementia, chronic obstructive lung disease.

**Results:**

501,121 individuals died in 2015, 59% of whom were in a hospital at the time of death. The aggregated spending totaled 9% of total health expenditures, a mean of €28,085 per capita, 44% of which was spent during the last 3 months of life. Hospital admissions represented over 70% of total expenditures; 21.3% of the population used hospital palliative care services in their last year of life. Analyses performed on breast cancer, dementia and lung disease found that differences in care pathways markedly influenced spending and were not simply explained by patients characteristics.

**Conclusion:**

Diagnoses and care trajectories, including repeated hospital stays, are the main drivers of the last year of life expenditures. Our data suggests that early identification of patients requiring palliative care and community-based end-of-life service delivery is feasible and could better support patients, families and caregivers with constant or reduced costs.

## 1. Introduction

End-of-life care could potentially be provided more efficiently, thereby improving the experience patients families and caregivers.^[1]^ Community-based specialist teams providing care in patients homes may be preferred to hospital care by both patients and families and could be cost reducing for the healthcare system.^[2,3]^ Estimates of end-of life costs and analysis of care pathways at a national level are needed in order to plan and budget alternative service delivery.^[4]^ In 2008, an estimate for France reported that end-of life care represented 10.5% of yearly health expenditures.^[5]^ An international comparison in 2017 found that spending during the last 12 months of life ranged from 8.5% (USA) to 11.2% (Taiwan) of aggregate spending.^[1]^ Authors also found that, although spending during the last 12 months of life was not wasteful, the money could be expended to provide better care. With the same focus on how to better deliver health services for end-of life care, we estimated healthcare spending over the last 12 months and the last 3 months of life, assessed the composition of services over the last year of life, and examined the differences in spending related to the main diagnoses and care trajectories for dementia, breast cancer and lung disease.

## 2. Patients and methods

### 2.1. Data

Our analysis was based on individual-level medical expenditures, using the national claims database for French citizens.^[6]^ The *système national d’information interrégimes de l’Assurance Maladie* [national health insurance information system] covers almost the entire population and all of their health care claims. The *système national d’information interrégimes de l’Assurance Maladie* (national claims database) data warehouse comprises characteristics and medical information of beneficiaries, as well as in-hospital or office medicine health care reimbursed to this population.

The type and date of procedures performed by health professionals, at the patient’s home, in private clinics or in health or medical and welfare centers are recorded. All outpatient medical and surgical procedures or imaging are coded as well as reimbursable drugs, or the use of homecare services and transport for medical purposes. Hospital admissions are coded using a diagnosis-related group system. A specific code is used for admission in palliative care units.

### 2.2. Study population

This retrospective observational study concerned all patients aged over 1 year deceased in 2015 from nonviolent or nonvoluntary death were included and their use of medical services recorded within 12 months of death.

### 2.3. Patient characteristics

For each patient, demographics, Charlson comorbidity index and health conditions were documented in the 12 months prior to the date of death. Health conditions (grouped in 9 categories) were identified by algorithms combining inpatient diagnoses international classification of diseases 10th revision code, long-term disease code used by the social health insurance, pharmacy, laboratory tests, and medical procedures reimbursement claims. The detailed methodology of the Diseases Mapping algorithms are publicly available in French.^[7]^

### 2.4. Outcomes

#### 2.4.1. Healthcare resource and costs.

Healthcare resources and their related costs over the twelve months before death were measured using data from both 2015 and 2014. Ambulatory care included general practitioner and specialist visits, paramedical fees (such as nurses and physiotherapists), pharmacy, laboratory tests, and certain direct nonmedical costs (e.g., medical devices and transportation services). Hospital care expenditures comprised expenditures related to acute hospitalization, rehabilitation, hospital at-home, psychiatry and home care. The use of hospital palliative care services was identified. Hospital expenditures were calculated for each stay from each diagnosis-related group. Expensive hospital drugs were added to the diagnosis-related group cost using the list price. All costs were inflated to current values.

### 2.5. Care pathways

Three conditions were selected for clustering analysis: breast cancer, dementia and obstructive lung disease, because they require the full range of care resources and are examples of cancer, degenerative and chronic conditions for which we expected that a shift from hospital to home care delivery would be possible.

Care pathways were defined as sequences in time of healthcare utilization and measured in the year before the death. The main variable consisted of a classification (typology) of care pathways by type of care: acute hospitalization, rehabilitation, hospital at-home, psychiatry, and home care (including homecare, community care, and nursing homes).

### 2.6. Statistical analysis

We first described the patients population in terms of age, sex, diagnosis, and Charlson index recorded at 12 months before death. Second, total expenditures were computed over the last twelve months and the last 3 months, by diagnosis, and with a focus on the highest expenditure decile.

Finally, we analyzed care pathways for breast cancer, dementia and chronic obstructive lung disease (COLD) by using a state sequence analysis.^[8]^ This method was specifically developed to analyze sequential data.^[9,10]^ Sequence analysis focuses on the evolution of a variable over a given period of time. The value taken by the variable at a given time t is called “state.” States represented the places of care such as acute hospitalization, rehabilitation, hospital at-home, psychiatry, and home care (including homecare community care and nursing homes). A “sequence” was defined as the succession of “daily states” during the 365 days preceding death. A care pathway was defined as the succession of sequences. Given the significant resources required to process information from the TraMiner package, sequence analysis was undertaken on representative samples of 10,000 patients randomly drawn from each population.^[11]^ Once the pathways were created, the sequence analysis compared them 2 by 2. We used Optimal Matching to measure the dissimilarity between 2 sequences by transforming 1 into the other using 3 elementary operations: insertion, deletion and substitution.^[11–14]^ The insertion (addition) and deletion (suppression) of a state have been brought together in the same concept of indel, formed by the contraction of the 2 names. Substitution replaces a state by another different state at the same time t. Each operation (indel and substitution) is assigned a specific cost that may be constant or may vary according to the states.^[12]^ We defined the measure of dissimilarity using the Longest Common Subsequence method by considering that the cost of an indel is equal to 1 and that the substitution cost is equal to 2.^[11]^ The dissimilarity matrix thus constructed was then used to classify the sequences in order to create the typology using Hierarchical Ascending Classification. We carried out the grouping of the classes using Ward method.^[10,12]^ One of the advantages of sequence analysis is the set of graphical representations available to describe and interpret the results of the classification into clusters. We used plot indexes and chronograms. Once each patient was classified in a specific cluster with similar care pathways, we analyzed individual characteristics and the care consumption between groups using the usual descriptive statistics.^[13]^ State sequence analysis was performed using the TraMineR package in R [43]. All other analyses were performed using SAS 9.4.

### 2.7. Ethics and authorization

The study was approved with access to fully anonymized patient-level data granted through Comité éthique et scientifique pour les recherches, les études et les évaluations dans le domaine de la santé (Ethics committee for healthcare research) and commission nationale informatique et libertés (national data safety commission) authorization, granted respectively in December 2018 and in February 2019.

## 3. Results

### 3.1. Patient characteristics

**Figure 1.**
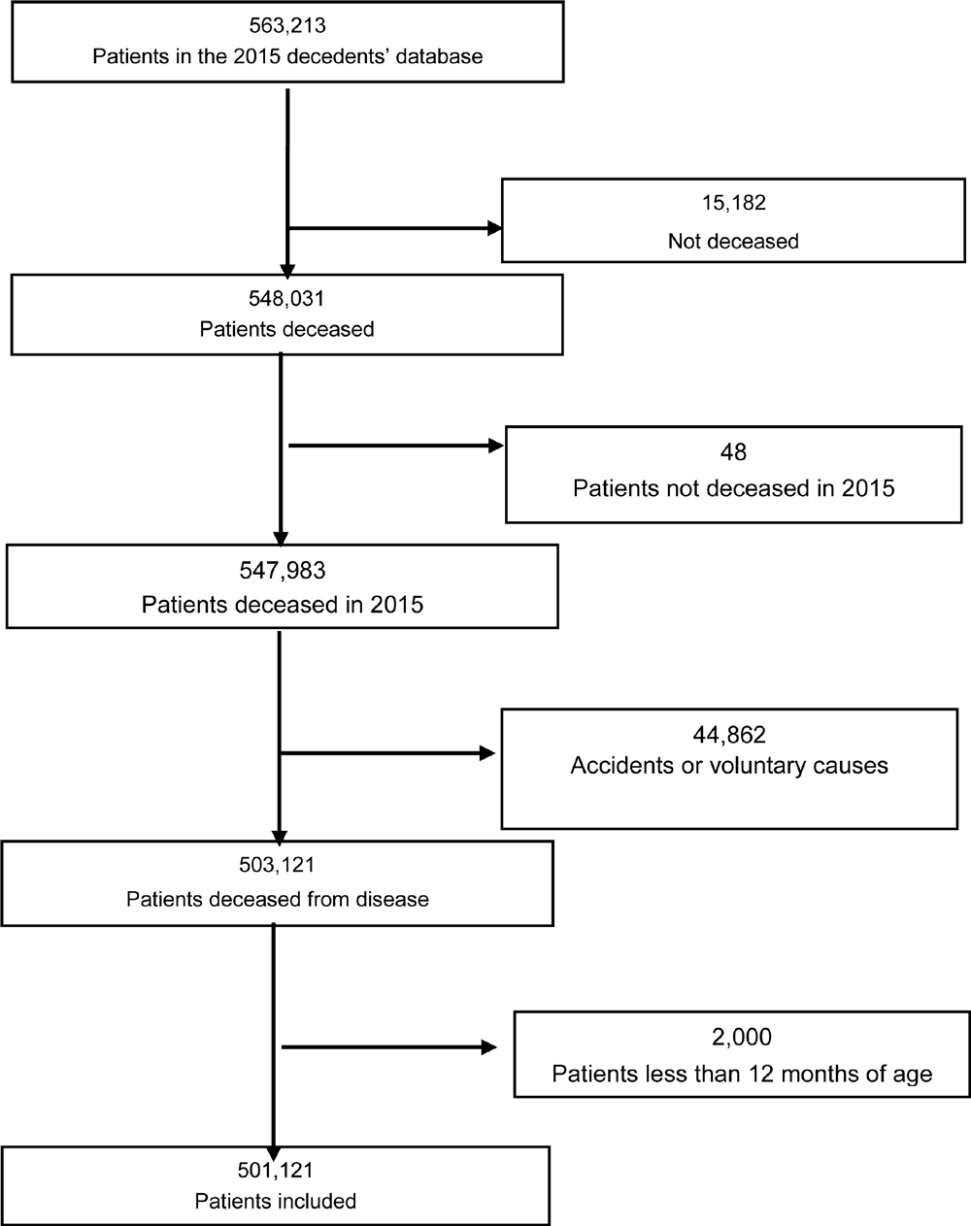
Flow chart of the patients selection.

Five lakhs and one thousand one hundred twenty-one individuals who died in 2015 were included (Fig. 1). Patients characteristics are presented in Table 1. The mean age at the time of death was 78.5 (±14.9) years and the sex ratio 0.97, mean Charlson index was 4.35 ± 3.76. Deaths before the age of 80 years predominantly concerned men, with a marked sex difference between the ages of 20 and 29 years.

**Table 1:** Characteristics of patients and diagnostic classification for the total population of 501,121 patients deceased in 2015 in France.

Socioeconomic characteristics	
Sex N (%)	
Male	247,440 (49.4)
Female	253,681 (50.6)
Age (yr) at death	
Mean (SD)	78.5 (14.9)
Age group (yr) at death N (%)	
<20	2452 (0.5)
[20 ;30]	3233 (0.6)
[30 ;40]	4310 (0.9)
[40 ;50]	12,632 (2.5)
[50 ;60]	34,089 (6.8)
[60 ;70]	64,632 (12.9)
[70 ;80]	83,809 (16.7)
[80 ;90]	177,204 (35.4)
>=90	118,760 (23.7)
Social deprivation index N (%)	
1st quartile (least deprived)	62,985 (16.0)
2nd quartile	63,884 (16.3)
3rd quartile	74,970 (19.1)
4th quartile	78,678 (20.0)
5th quartile (most deprived)	86,095 (21.9)
Missing	26,198 (6.7)
Charlson comorbidity index	
Charlson score	
Mean (SD)	4.3 (3.7)
Charlson score per class N (%)	
0	43,613 (8.7)
1–2	169,514 (33.8)
3–4	109,643 (21.9)
≥5	178,351 (35.6)
Diagnoses in the 12 months prior to the date of death N (%)	
Dementia	95,409 (19.0)
Chronic obstructive lung disease (COLD)	86,540 (17.3)
Breast cancer	11,311 (2.3)
Lung cancer	25,087 (5.0)
Prostate cancer	11,373 (2.3)
Acute stroke	21,466 (4.3)
Acute coronary syndrome	7310 (1.4)
Parkinson disease	17,380 (3.5)
End-stage renal failure and ongoing kidney dialysis	6180 (1.2)
Identified long-term illness N (%)	409,525 (82%)
Eligibility for state-sponsored health insurance N (%)	30,309 (7.7%)

The selected diagnoses do not add up to 100%.

### 3.2. Health care resource use and place death

During the year preceding death, 85,2% patients were hospitalized at least once, 92% of patients visited a general practitioner or other professionals. Overall, 59% of deaths occurred in a hospital (49% in acute care, 7% in rehabilitation, 3.2% with hospital at home services).

### 3.3. Use of hospital palliative care

Hospital palliative care services were used by 135 702 patients or 27% of the population. This proportion varied according to sex (men: 29%; women: 25%), but also according to age, with a peak around 38% between the ages of 50 and 70 years (Table S1, Supplemental Digital Content, http://links.lww.com/MD/J431 presents the characteristics of patients receiving palliative care).

### 3.4. End-of life expenditures

Only 5% of the cohort population did not incur any medical spending during the 12 months preceding death. Total expenditures in the last 12 months of life represented 14 billion € or 9% of the total healthcare spending in France. Mean per capita medical spending in the last twelve months and the last 3 months of life reached €28,085 and €12,385 (or 44% of the 1-year total) respectively. A detailed description of expenditures by service category is presented Table 2A. Palliative care in hospitals accounted for 19% of the hospitalization costs (2833 €). When restricting the analysis to the population of patients who received palliative care (27% of the population), the average 12-month cost was 26,324 € (lower than the average), representing 25% of all healthcare expenditure.

**Figure 2.**
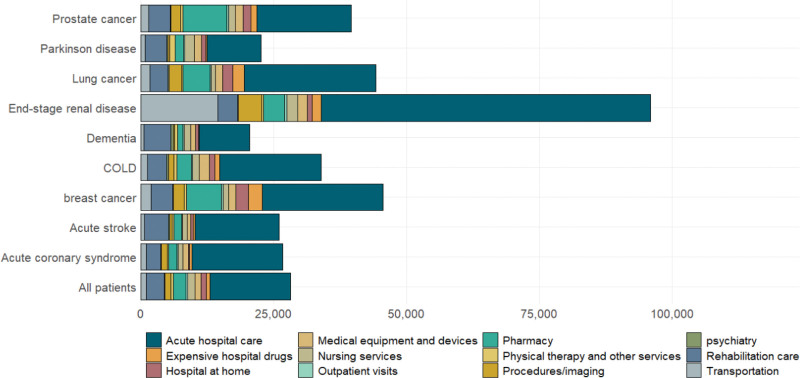
Last 12-month expenditures by service type for 9 major diagnoses. 12-month end-of life expenditures by disease (diagnoses coded during the last 12 months).

The most expensive diagnostic categories were tumors, both hematological and solid. For all diagnostic categories, acute hospital care represented the highest proportion of expenditures. Patients with end-stage renal failure and ongoing kidney dialysis incurred the highest expenditures for an average of €97,693 (Fig. 2). Expenditures by service type for the 3 diagnoses selected for the cluster analysis are presented Table 2B; Table S2, Supplemental Digital Content, http://links.lww.com/MD/J432 presents expenditures for the subgroups of patients receiving palliative care in these selected diagnoses.

### 3.5. Characteristics of high-cost patients

Among the 501,121 patients deaths in 2015, the 50,113 in the highest decile had medical expenditures above €65,914 and accounted for 37.5% of total expenditures. These individuals were younger (70 vs 79 years), less often female (41.7% vs 51.6%), and had more severe comorbidities (Charlson index: 8 vs 4) than the rest of the population.

### 3.6. Care pathways and cluster analysis

A total of 11,311 breast cancer patients (2.3% of total deaths), 95,409 dementia patients (19.0%) and 86,540 (17.3%) COLD patients were entered in the cluster analyses. Three types of clusters were identified, and labeled: “few and late hospitalizations” (cluster 1); “acute care during the last 3 months of life” with alternating rehabilitation and acute care (cluster 2); “early and repeated hospitalizations” with repeated use of hospital at-home, rehabilitation and acute care (cluster 3). The proportion of patients in each cluster type differed by disease. Costs by disease and by cluster are presented in Table 2C.

### 3.7. Patients with breast cancer and patients with dementia

Summary statistics of patient characteristics by clusters are presented in Table S3 A–C, Supplemental Digital Content, http://links.lww.com/MD/J433. Table S1 A and B, Supplemental Digital Content, http://links.lww.com/MD/J434 present the state distribution plots of the breast cancer and dementia patients, respectively. The majority of patients with dementia and breast cancer were found in the cluster 1with few and late hospitalizations (67% of patients with dementia and 58% of breast cancer patients), while the cluster 2 with acute care during the last 3 months comprised 27% dementia and 36% breast cancer patients.

The breast cancer women in cluster 1 were older (69.6 ± 14.7 years) than the cluster 3 (61.9 ± 14.1 years) and slightly older than the cluster 2 (68.5 ± 14.1 years), had fewer severe comorbidities (Charlson index: 8.9 ± 2.6) than in the 2 other clusters (9.6 ± 2.1 and 10.0 ± 2.0 for cluster 2 and 3, respectively). The mean costs per patient varied considerably between groups of pathways. Cluster 3 represented 10% of total cost and only 5% of the population (Table 2C). The majority of total costs resulted from other clusters (47% and 43% of total costs for clusters 2 and 3, respectively).

Results on dementia patients showed that the patients in cluster 1 were slightly older (86.1 ± 7.1 years) than in the 2 other clusters (84.6 ± 7.9 and 83.8 ± 8. for cluster 2 and 3, respectively), more often female (62.5% vs 56.7% and 56.1% for cluster 2 and 3, respectively), and had fewer severe comorbidities (Charlson index: 3.4 ± 2.5 vs 4.9 ± 3.3 and 5.6 ± 3.6 for cluster 2 and 3, respectively). The mean costs per patient varied considerably between clusters of pathways. The mean costs were nearly 4.75-fold higher in the cluster 3 compared to the cluster 1. The cluster 3 represented 17.5% of total costs, despite the group’s small size (6.0%).

### 3.8. Patients with COLD

Patients with COLD were grouped into 2 clusters, 84% had few or late hospital admissions and 16% had early, recurrent and long acute care hospital stays. Figure S1 C, Supplemental Digital Content, http://links.lww.com/MD/J434 presents the state distribution plot. Patients in cluster 1 were older (77.2 ± 12.9 years vs 72.2 ± 13.2 for cluster 2), more often female (41.2% vs 37.2% for cluster 2) and had fewer severe comorbidities (Charlson index: 5.5 ± 3.6 vs 7.9 ± 3.9 for cluster 2). The contribution to the total costs was notable (34%) for cluster 2, despite the group’s small size (15.9% of the population).

Table S4 A–C, Supplemental Digital Content, http://links.lww.com/MD/J435 present the odds ratios for factors associated with care pathways in each disease.

## 4. Discussion

Total end-of life expenditures were 14 billion € or 9% of the total healthcare spending in France. Our results show that acute hospital care was the main cost driver, representing 54% of total spending, whatever the main diagnosis class, and that spending of the last year of life markedly differed between diagnosis classes. There is a growing trend in end-of life expenditures per patient which represented 22,000 € (2015 current €)/patient in 2008 and 28 000 € in 2015 (5).

For dementia, breast cancer or COLD, care pathways and costs differed widely as shown in our cluster analyses. A key finding is that patients in the least desirable care pathway, with early and repeated admissions during the last year of their lives, represented fewer than 20% patients in all 3 diagnosis categories. This care pathway was also associated with much higher costs in the 3 diseases as compared to “few or late hospitalizations” or “acute care during the last 3 months of life” trajectories. Variables associated with the likelihood of being in the “early and repeated admissions” pathway suggested that patients characteristics played a relatively small role; we hypothesize that supply-induced demand, whereby the pattern of care of the last months of life repeats the earlier use of services, has a part in defining patients trajectories.

Based on projections from the National Institute of Demographic Studies, we know that number of deaths within French population will increase up to 770,000 deaths in 2050, which represents a + 30% growth compared to latest data. This dramatic increase will pressure heath system and health expenditures. Offering patients and families more end-of-life care options, and encouraging palliative care at home could be achieved at constant or reduced spending.^[15–18]^ Community-based palliative could help caregivers identifying individuals with chronic conditions and functional limitations^[19]^ and initiating the use of home services early, thus answering patients will to stay at home as long as possible (a common figure being 80% preferring to die at home).

We saw high users of hospital services represent only 10% to 15% of the total number of decedents and therefore reaching out to them is feasible, with the suggestion that these patients could benefit from palliative care and improved end-of-life healthcare allocation.^[15,20]^ Palliative care in general has been found to reduce hospital days and hospital costs, early palliative care has been found to reduce hospital days compared to late palliative care: patients in the most expensive cluster (early and frequent admissions) could also benefit from an early management as they are admitted early. This patient population had a high Charlson index, which was also found to be associated with a greater benefit from palliative care.^[21,22]^ Palliative care teams outside hospitals could be substituted to hospital care with a high level of satisfaction from families; however it is underdeveloped in France, as only hospital services are currently listed on the official reimbursement schedule.^[16]^ Ongoing experiments of bundled payment allowing reimbursement for unlisted services might remove some of the barriers to the development of integrated pathways, but current organization is hospital-centered for historical reasons.

Similar patterns of service use and expenditures were found in other countries, regarding the share of total health expenditures and the differences by place of death^[17–19]^ with calls for organized end-of life care that allows patients who wish it to remain at home.^[23–25]^

In France, end-of life care (palliative), initially developed for patients with cancer, was established as a national priority by ministerial plans since 1999. The latest plan (2015–18) emphasizing the need for palliative care at home was insufficiently deployed, with a limited involvement from general practitioners.^[26]^ Among weaknesses and shortcomings identified for empowering general practitioners were the lack of training, quasi disappearance of home visits in the medical practice, lack of financial incentives, and insufficient support from palliative care networks. Self-employed nurses who visit patients at home also lack training and support. This failure to provide appropriate supply of professionals at the community level meant that palliative care was finally entrusted to hospitals, with a high financial and emotional cost.

Community-led palliative care requires adequate human resources and financing mechanisms.^[24,27]^ A proposed French experiment is to train volunteers who work at patients homes in collaboration with healthcare professionals. The role of general practitioners would be mostly to identify patients who could benefit from early intervention of palliative care teams with fewer hospital admissions. The proposed financial mechanism to encourage the shift toward community-led care is an accountable care organization-like system whereby a capitated weekly fee would cover a required estimated 0.6 full time equivalent for 120 patients a year, supplemented by volunteers and piloted by healthcare professionals also paid a capitated fee.

The strength of our study is that we used a national, comprehensive database, from a single payer system which included every health service, regardless of reimbursement. Limitations are that our analysis was descriptive, non-interventional, and did not provide information on the quality of care or patients experience. Our analysis is claims based and does not include spending outside the healthcare system (informal care) or healthcare resources for which no claim was sent. The French claims database groups together patients living at home and nursing home residents, who might have different needs and different healthcare utilization patterns regarding end-of life care. We did not undertake a separate analysis in younger patients because they have specific needs and requirements that are likely to differ from end-of-life care in elderly patients.

The major limitation pertains to the year of the data. As the Covid period would not be representative of end-of-life treatment, we examined the location of death, as a proxy for end-of-life care, between the years 2016 to 2019. In 2015, the percent of patients who died in the hospital was 59% in our sample (to which 3.2% with hospital at home services should be subtracted) and 55. 9 % using the INSEE (national statistics) data, the INSEE percentage was 53.5 in 2018 and 53% in 2019.^[28]^ The place of death that increased over the 2015 to 2019 period is “other” which covers the workplace, roads and missing data. The time series in the INSEE report shows stability since 2010 in the percent of hospital deaths up to 2019, the Covid period of 2020 to 2022 is analyzed separately. Inflated with the national index for healthcare, the total expenditure in 2019 would be 15.2 billion €.

## 5. Conclusion

Understanding healthcare use and care pathways at the end-of life allows planning for alternative methods of care delivery. Planning better end-of life care that limits the use of hospital could be possible if an early identification of high users patients for whom home care is feasible. Limiting the use of acute care will not automatically reduce costs, but could ensure better use of hospital services and better respect for patients and families expectations.

**Table 2A:** Last 12-month expenditures for patients deceased in 2015, by service category, in € for the entire population N = 501,121.

Service type	% users	Mean, cost per patient (SD)	(% of total expenditure)	Median (IQR)
Acute hospital care	76%	15,111 (22,537)	54	8218 (18,387)
Rehabilitation care	21%	3496 (16,894)	12	0 (0)
Hospital at home	6.3%	986 (8265)	4	0 (0)
Nursing services	66%	1297 (3158)	5	30 (586)
Outpatient visits	77 %	308 (307)	1	248 (328)
Physical therapy and other services	42%	406 (862)	1	0 (364)
Medical equipment and devices	76%	1178 (2770)	4	216 (1323)
Pharmacy	89%	2409 (6580)	9	811 (1535)
Expensive hospital drugs	9%	765 (20,158)	3	0 (0)
Transportation	82%	992 (2859)	4	270 (822)
Procedures/imaging	70%	1137 (2606)	4	332 (979)
Total	95%	€28,085 (35,895)		16,875 (31,657)

Other services include: psychiatric services, biology and imaging.

Cost of medical devices include the services related to the functioning of these devices.

95% of decedents used at least one type of healthcare service during the last 12 months of their lives.

**Table 2B:** Mean, standard deviation, median and IQ range for 12-month expenditures in € for: dementia, breast cancer, and chronic obstructive lung disease. Analyses were performed on a random sample of 10,000 patients.

Service type	Dementia		Breast cancer		Chronic obstructive lung disease	
	Mean (SD)	Median (IQR)	Mean (SD)	Median (IQR)	Mean (SD)	Median (IQR)
Acute hospital care	9434 (13,765)	6551 (12,743)	22,754 (22,806)	17,316 (24,629)	19,141 (24,845)	14,887 (21,392)
Rehabilitation care	4998 (19,361)	0 (0)	4095 (15,567)	0 (0)	3682 (15,894)	0 (0)
Hospital at home	516 (5540)	0 (0)	2324 (12,155)	0 (0)	1036 (7282)	0 (0)
Nursing services	1294 (3404)	6 (769)	1060 (3797)	156 (801)	1247 (3346)	109 (1128)
Outpatient visits	280 (295)	253 (309)	381 (245)	331 (386)	354 (341)	322 (331)
Physical therapy and other services	616 (1099)	55 (998)	391 (748)	73 (516)	498 (971)	52 (624)
Medical equipment and devices	848 (1879)	124 (1102)	1300 (3527)	428 (1381)	1908 (3742)	891 (2797)
Pharmacy	998 (2398)	690 (954)	6650 (17,079)	3336 (7671)	2764 (6898)	1428 (1996)
Expensive hospital drugs	177	0 (0)	2658	0 (0)	808	0 (0)
Transportation	614 (2065)	304 (571)	1185 (2910)	1016 (2332)	1864 (1367)	514 (1129)
Procedures/imaging	940 (1443)	238 (499)	3404 (3407)	1456 (2850)	1142 (3743)	536 (2151)
Total	€20,715 (29,435)	13,938 (22,693)	€46,202 (39,682)	39,041 (40,626)	€34,444 (35,884)	28,431 (34,745)

**Table 2C:** Costs for the cluster analysis of dementia, breast cancer and chronic obstructive lung disease (COLD). The population in each cluster was a random sample of 10,000 individuals drawn from the total population of patients with the corresponding diagnosis.

	Dementia		Breast cancer		COLD	
	Mean (SD)	Median (IQR)	Mean (SD)	Median (IQR)	Mean (SD)	Median (IQR)
Cluster 1 = few/ late hospitalizations	N = 6725 €16,139 (€15,379)	€12,588 (€12,926)	N = 5848 €35,872 (€33,586)	€30,771 (€30,161)	N = 8410 €30,512 (€26,001)	€24,304 (€26,207)
Cluster 2 = acute care during the last 3 months of life	N = 2664 €42,045 (€31,991)	€34,819 (€28,199)	N = 3609 €62,643 (€37,709)	€55,288 (€40,664)	NA	NA
Cluster 3 = early and repeated hospitalizations	N = 611 €76,759 (€54,026)	€64,352 (€51,294)	N = 543 €91,246 (€46,947)	€81,783 (€55,381)	N = 1590 €82,164 (€47,747)	€71,333 (€51,821)

## Author contributions

**Conceptualization:** Daniel Szfetel, Bénédicte Denoyel, Omar Beloucif, Joelle Texereau, Louis Champion, Jean François Vié, Isabelle Durand-Zaleski.

**Data curation:** Arnaud Nze Ossima.

**Formal analysis:** Arnaud Nze Ossima.

**Funding acquisition:** Bénédicte Denoyel, Omar Beloucif, Louis Champion, Jean François Vié.

**Methodology:** Arnaud Nze Ossima, Daniel Szfetel, Isabelle Durand-Zaleski.

**Supervision:** Daniel Szfetel, Jean François Vié.

**Validation:** Arnaud Nze Ossima, Daniel Szfetel, Bénédicte Denoyel, Joelle Texereau, Louis Champion, Jean François Vié.

**Writing – original draft:** Arnaud Nze Ossima, Isabelle Durand-Zaleski.

**Writing – review & editing:** Arnaud Nze Ossima, Daniel Szfetel, Bénédicte Denoyel, Omar Beloucif, Joelle Texereau, Louis Champion, Jean François Vié, Isabelle Durand-Zaleski.

## Supplementary Material










