# Supplementary material for: End-of life medical spending and care pathways in the last 12 months of life: A comprehensive analysis of the national claims database in France
Source: Medicine (Baltimore). 2023 Aug 4;102(31):e34555. doi: 10.1097/MD.0000000000034555 (PMC10403027; doi:10.1097/MD.0000000000034555)
Supplement: Supplementary file 1 [file medi-102-e34555-s001.pdf]

Table S1 Patients receiving palliative care (N= 135 702 patients) in a hospital setting; the total population is N= 501,121

	Any type of hospital	Acute care hospital	Réhabilitation hopital	Hospital at home
	%	%	%	%
Total N= 501,121	27.08	22.52	4.75	4.47
Men N= 247,440	29.13	24.62	4.66	4.84
Women N= 253,681	25.08	20.46	4.83	4.10
Age : % of patients in each age group receiving palliative care (N)				
<20 N= 455	18.57	17.59	0.65	5.77
[20 ;30[N=353	10.92	10.15	0.40	3.19
[30 ;40[N= 1,071	24.85	22.95	1.39	6.77
[40 ;50[N=4,156	32.90	30.14	2.71	7.44
[50 ;60[N=12,985	38.09	34.66	4.20	7.42
[60 ;70[N=24,896	38.52	34.26	4.95	6.93
[70 ;80[N=28,822	34.39	29.22	5.78	5.90
[80 ;90[N=43,699	24.66	19.51	5.40	3.55
>=90 N=19,263	16.22	12.00	3.64	2.24

State sponsored complementary insurance	30.77	27.50	3.97	5.79
N= 30,309 patients				

Out of the total population of 135,702 patients receiving palliative care, 72,079 were men and 63,623 were women. For age groups, the percentages correspond to the part of the population in each age group receiving palliative care, for example out of the 177,204 patients aged 80-90 years who died, 43,699 had received palliative care (or 24.66% of the total population in the age group) and the share by type of palliative care is described in the subsequent cells in line. The same patient could receive palliative care in several types of hospital, therefore the total of percentages in the 3 last columns can be superior to the cell in the first column.
