# Supplementary material for: End-of life medical spending and care pathways in the last 12 months of life: A comprehensive analysis of the national claims database in France
Source: Medicine (Baltimore). 2023 Aug 4;102(31):e34555. doi: 10.1097/MD.0000000000034555 (PMC10403027; doi:10.1097/MD.0000000000034555)
Supplement: Supplementary file 2 [file medi-102-e34555-s002.pdf]

Table S2

Last 12-month expenditures in € per capita for the selected diagnoses (dementia, breast cancer, and chronic obstructive lung disease) **restricted to patients with use of palliative care.**

Service type	Dementia N=2,575	Breast cancer N=6,731	Chronic obstructive lung disease N= 3,262
Acute hospital care	10,885	15,856	15,037
Rehabilitation care	5,759	5,900	4,683
Hospital at home	505	1,159	696
Nursing services	1,526	1,459	1,609
Outpatient visits	329	426	397
Physical therapy	720	548	674
Medical equipment and devices	948	1,228	1,807
Pharmacy	1,137	3,333	1,929
Expensive hospital drugs	181	695	393
Transportation	681	1178	934
Procedures/imaging	871	1801	1313
**total**	**€23,544**	**€33,582**	**€29,471**
