# Supplementary material for: End-of life medical spending and care pathways in the last 12 months of life: A comprehensive analysis of the national claims database in France
Source: Medicine (Baltimore). 2023 Aug 4;102(31):e34555. doi: 10.1097/MD.0000000000034555 (PMC10403027; doi:10.1097/MD.0000000000034555)
Supplement: Supplementary file 3 [file medi-102-e34555-s003.pdf]

Table S3 A Characteristics of patients with dementia by cluster. Analyses were performed on a random sample of 10,000 patients. Patients were grouped into clusters with comparable care pathways.

	cluster 1= few/ late hospitalizations	cluster 2= acute care during the last 3 months of life	cluster 3= early and repeated hospitalizations	*Total patients*
Sex (%)Female	4204 (62.5)	1510 (56.7)	343 (56.1)	6057 (60.6)
Age (years) Mean (SD)	86.1 (7.1)	84.6 (7.9)	83.8 (8.1)	85.5 (7.4)
Charlson score	3.4 (2.5)	4.9 (3.3)	5.6 (3.6)	4.0 (2.9)

Table S3 B Characteristics of patients with breast cancer by cluster. Analyses were performed on a random sample of 10,000 patients. Patients were grouped into clusters with comparable care pathways.

	cluster 1=few/ late hospitalizations	cluster 2= acute care during the last 3 months of life	cluster 3= early and repeated hospitalizations	*Total patients*
Age (years) Mean (SD)	69.6 (14.7)	68.5 (14.1)	61.9 (14.1)	68.8 (14.5)
Charlson index Mean (SD)	8.9 (2.6)	9.6 (2.1)	10.0 (2.0)	9.2 (2.4)

Table S3 C Characteristics of patients with Chronic obstructive lung disease by cluster. Analyses were performed on a random sample of 10,000 patients. Patients were grouped into clusters with comparable care pathways.

	cluster 1= few/ late hospitalizations	cluster 3= early and repeated hospitalizations	*Total patients*
Sex (%)Female	3463 (41.2)	591 (37.2)	4054 (40.5)
Age (years) Mean (SD)	77.2 (12.9)	72.2 (13.2)	76.4 (13.1)
Charlson index Mean (SD)	5.5 (3.6)	7.9 (3.9)	5.9 (3.7)
