# Supplementary material for: End-of life medical spending and care pathways in the last 12 months of life: A comprehensive analysis of the national claims database in France
Source: Medicine (Baltimore). 2023 Aug 4;102(31):e34555. doi: 10.1097/MD.0000000000034555 (PMC10403027; doi:10.1097/MD.0000000000034555)

Figures S1 (A,B, C) cluster analysis for dementia, breast cancer and chronic obstructive lung disease

Figures S1 cluster analysis for dementia, breast cancer and chronic obstructive lung disease

Cluster type 1: few and late hospitalizations

Cluster type 2: acute care during the last 3 months of life

Cluster 3: early and repeated hospitalizations

Figure S1-A cluster analysis for patients with a primary diagnosis of dementia

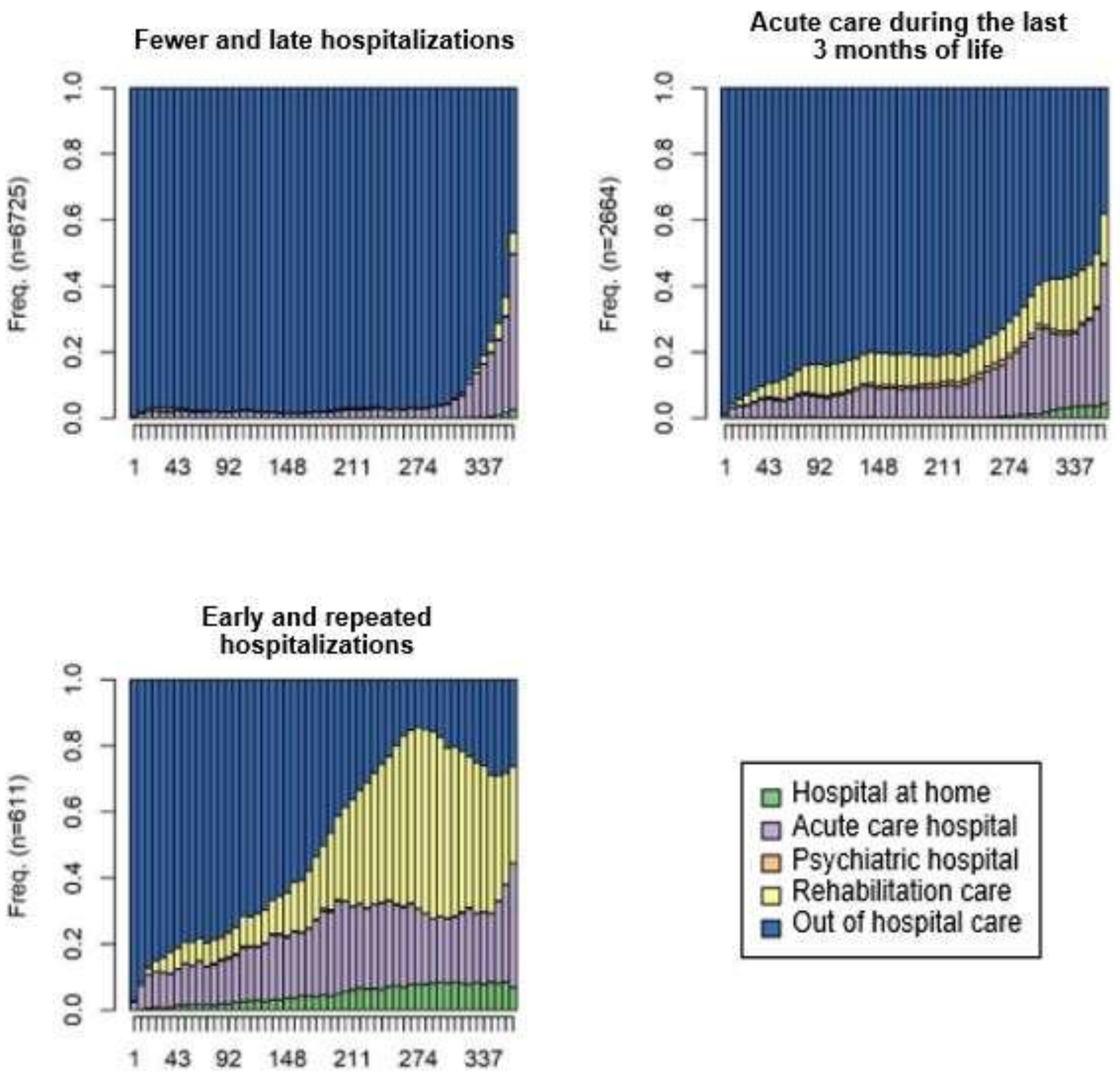

Figure S1-B cluster analysis for patients with a primary diagnosis of breast cancer

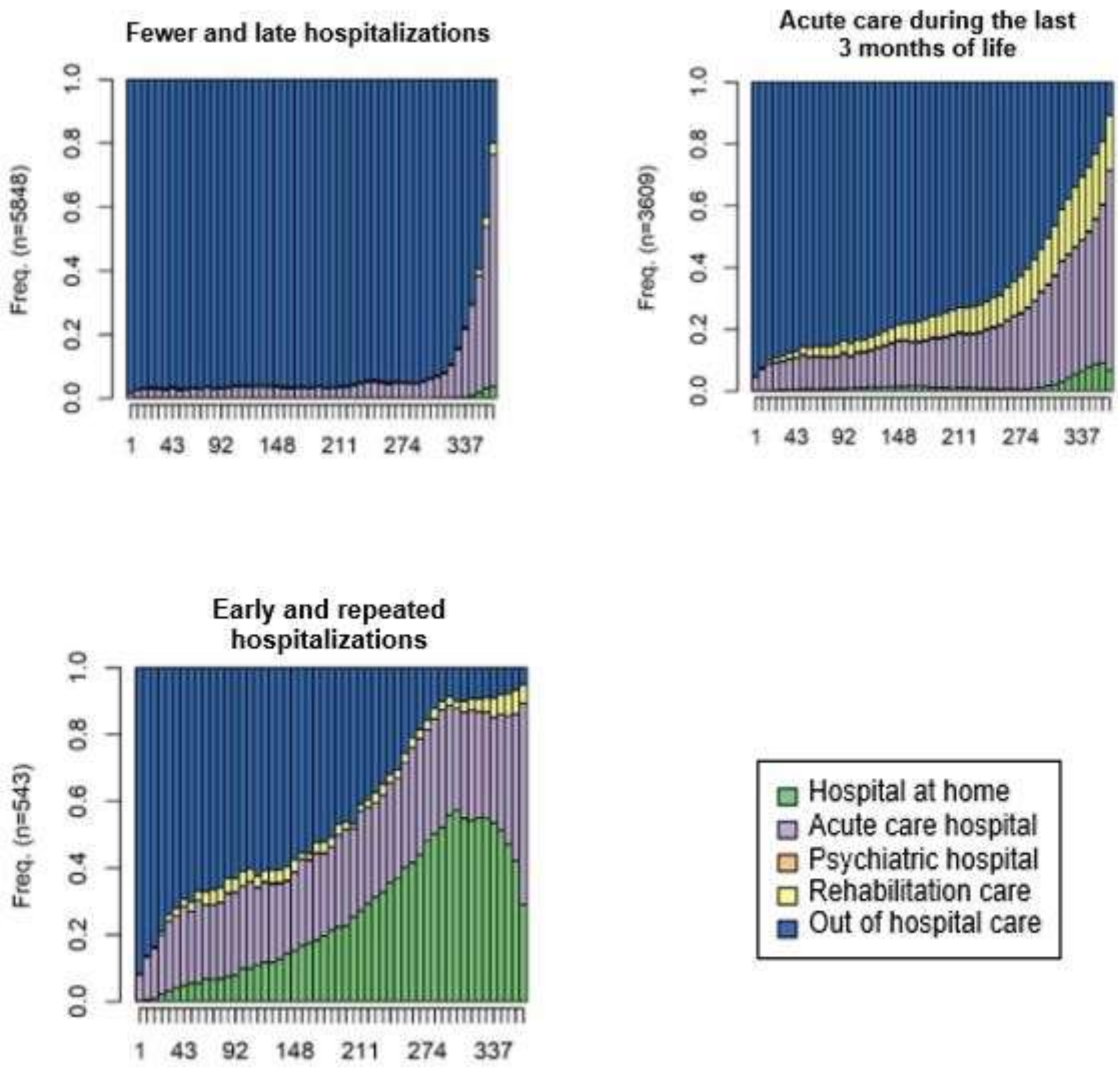

Figure S1C. Cluster analysis for patients with a primary diagnosis of chronic obstructive lung disease

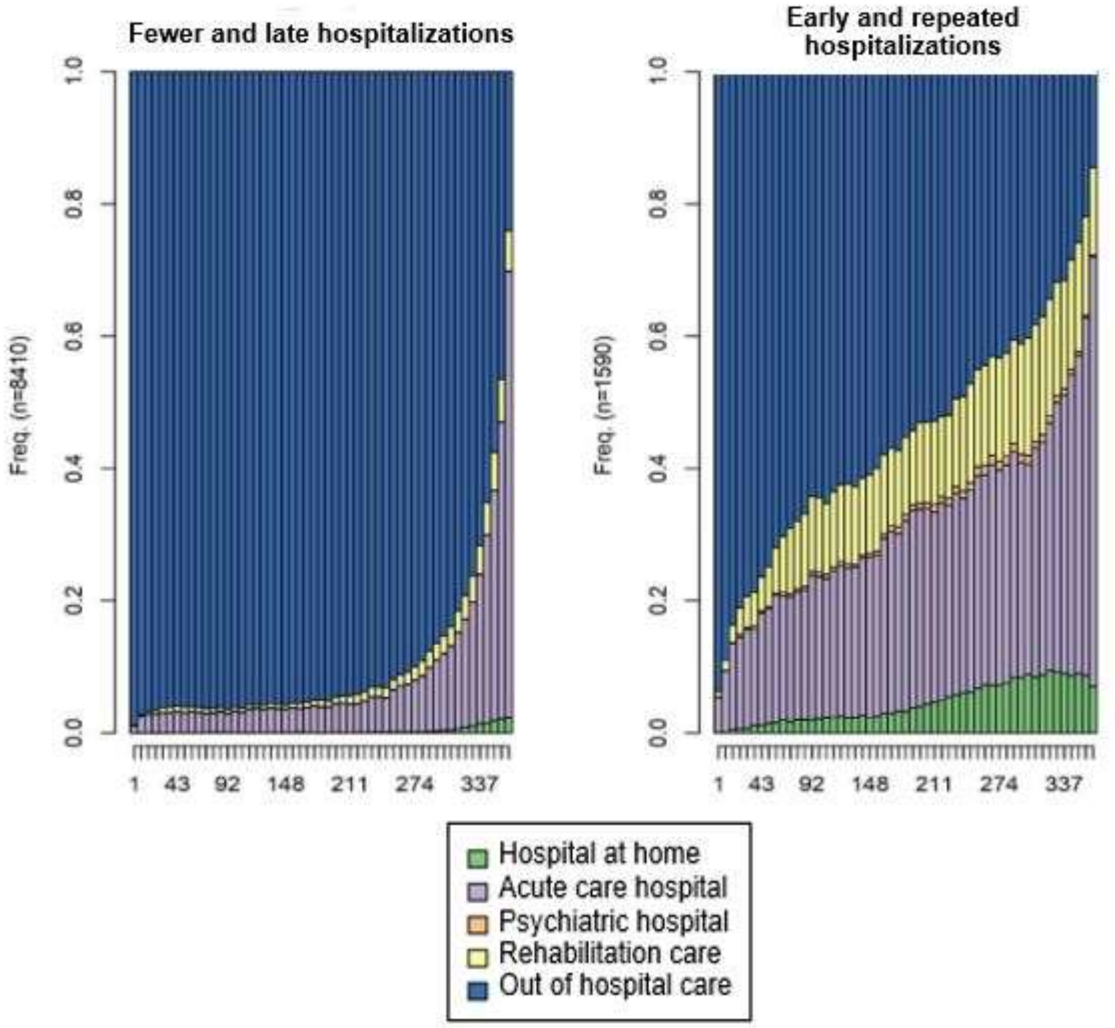

Supplement: Supplementary file 4 [file medi-102-e34555-s004.pdf]
