# Supplementary material for: End-of life medical spending and care pathways in the last 12 months of life: A comprehensive analysis of the national claims database in France
Source: Medicine (Baltimore). 2023 Aug 4;102(31):e34555. doi: 10.1097/MD.0000000000034555 (PMC10403027; doi:10.1097/MD.0000000000034555)
Supplement: Supplementary file 5 [file medi-102-e34555-s005.pdf]

Tables S4.

Factors associated with care trajectories in dementia, breast cancer, and chronic obstructive lung disease. The reference cluster chosen was ‘few/late hospital admissions’ (cluster type 1)

Table S4A Dementia

OR (95% CI) (ref: few /late hospitalizations, 67.25 % of the population)

	Acute care during the last 3 months of life (26.64 %) cluster type 2	Early and repeated hospitalizations (6.11 %) cluster type 3
Deprivation (Ref : Q1 les deprived quintile)	1.11 (0.99-1.25, p=0.06)	0.81 (0.78-0.84)
Age (+ 1 year)	0.99 (0.98-0.99)	0.98 (0.98-0.98)
Charlson index	1.06 (1.04-1.09)	1.07 (1.01-1.11)
Hospital transfer	3.47 (3.09-3.89)	4.83 (4.65-5.03)
Use of hospital palliative care	1.43 (1.27-1.59)	2.50 (2.41-2.58)
Use of home care (hospital at home)	1.67 (1.50-1.87)	1.71 (1.67-1.74)
Use of outpatient hospital care	1.31 (1.28-1.33)	1.49 (1.48-1.50)
Emergent hospital admissions 6-9 months before death	1.74 (1.62-1.87)	2.13 (2.09-2.17)
Hospital admission 9 months before death	2.57 (2.31-2.87)	3.02 (2.92-3.13)
Hospital admissions 6-9 months before death	3.43 (3.23-3.64)	13.67 (13.61-13.72)
Hospital admissions 3months before death	1.89 (1.77-2.01,)	2.78 (2.73-2.83)
Direct (scheduled) Hospital admission 6-9 months before death	1.88 (1.71-2.06,)	2.31 (2.25-2.38)

All p values are <0.001

Table S4 B: Breast cancer OR (95% CI) (ref: few /late hospitalizations, 58.48 % of the population).

All p values are <0.001

	Acute care during the last 3 months of life (36.09%) cluster type 2	Early and repeated hospitalizations (5.41%) cluster type 3
Age (+ 1 year)	1.006(1.004-1.008)	0.981(0.976-0.986)
Charlson index	1.042(1.022-1.062)	1.041(1.008-1.075)
Hospital transfer	2.777(2.749-2.806)	2.098(2.095-2.101)
Use of hospital palliative care	2.217(2.129-2.309)	7.896(7.862-7.930)
Use of outpatient hospital care	2.002(1.926-2.081)	2.278(2.264-2.292)
Emergent hospital admissions 6-9 months before death	1.348(1.278-1.421)	1.36(1.349-1.371)
Hospital admission 9 months before death	1.798(1.775-1.822)	2.763(2.756-2.771)
Hospital admissions 6-9 months before death	3.033(2.859-3.218)	11.242(11.195-11.290)
Hospital admissions 3months before death	1.731(1.669-1.796)	1.335(1.327-1.344)
Direct (scheduled) Hospital admission 6-9 months before death	0.846(0.801-0.894)	0.600(0.594-0.605)

Table S4 C: Chronic obstructive lung disease

OR (95% CI) Ref : few/late hospitalizations, 84.10 % of the population.

	Early and repeated hospitalizations (15.9%)
Age (+ 1 year)	0.99 (0.98-0.99)
Charlson index	1.04 (1.02-1.06)
Hospital transfer	3.28 (2.76-3.90)
Use of hospital palliative care	1.91 (1.66-2.19)
Use of home care (hospital at home)	1.26 (1.05-1.53)
Use of outpatient hospital care	1.44 (1.23-1.70)
Emergent hospital admissions 6-9 months before death	1.62 (1.38-1.89)
Hospital admissions 9 months before death	2.98 (2.37-3.)
Hospital admissions 6-9 months before death	5.04 (3.93-6.50)

All p values are <0.001
